# Macrophage-Targeted Lung Delivery of Dexamethasone Improves Pulmonary Fibrosis Therapy via Regulating the Immune Microenvironment

**DOI:** 10.3389/fimmu.2021.613907

**Published:** 2021-02-18

**Authors:** Xiaoqing Sang, Yuanyuan Wang, Zhifeng Xue, Dawei Qi, Guanwei Fan, Fei Tian, Yan Zhu, Jian Yang

**Affiliations:** ^1^Tianjin Key Laboratory of Chinese Medicine Pharmacology, Tianjin University of Traditional Chinese Medicine, Tianjin, China; ^2^State Key Laboratory of Component-Based Chinese Medicine, Tianjin University of Traditional Chinese Medicine, Tianjin, China; ^3^Medcity Research Laboratory, University of Turku, Turku, Finland; ^4^Medical Experiment Center, First Teaching Hospital of Tianjin University of Traditional Chinese Medicine, Tianjin, China; ^5^Tianjin Key Laboratory of Translational Research of Traditional Chinese Medicine Prescription and Syndrome, Tianjin, China; ^6^Tianjin Key Laboratory of Traditional Chinese Medicine Chemistry and Analysis, Tianjin University of Traditional Chinese Medicine, Tianjin, China

**Keywords:** macrophages, pulmonary fibrosis, drug delivery, phenotypic regulation, immune microenvironment

## Abstract

Idiopathic pulmonary fibrosis (IPF) is serious chronic lung disease with limited therapeutic approaches. Inflammation and immune disorders are considered as the main factors in the initiation and development of pulmonary fibrosis. Inspired by the key roles of macrophages during the processes of inflammation and immune disorders, here, we report a new method for direct drug delivery into the *in-situ* fibrotic tissue sites *in vitro* and *in vivo*. First, liposomes containing dexamethasone (Dex-L) are prepared and designed to entry into the macrophages in the early hours, forming the macrophages loaded Dex-L delivery system (Dex-L-MV). Chemokine and cytokine factors such as IL-6, IL-10, Arg-1 are measured to show the effect of Dex-L to the various subtypes of macrophages. Next, we mimic the inflammatory and anti-inflammatory microenvironment by co-culture of polarized/inactive macrophage and fibroblast cells to show the acute inflammation response of Dex-L-MV. Further, we confirm the targeted delivery of Dex-L-MV into the inflammatory sites *in vivo*, and surprisingly found that injected macrophage containing Dex can reduce the level of macrophage infiltration and expression of the markers of collagen deposition during the fibrotic stage, while causing little systematic toxicity. These data demonstrated the suitability and immune regulation effect of Dex-L-MV for the anti-pulmonary process. It is envisaged that these findings are a step forward toward endogenous immune targeting systems as a tool for clinical drug delivery.

## Introduction

Idiopathic pulmonary fibrosis (IPF) is the most common form of idiopathic interstitial pneumonia of unknown etiology. There is currently poor prognosis with increased morbidity and prevalence (1). Currently, only two clinical approved drugs, nintedanib (NDN) and pirfenidone (PFD) are being pursued to treat mild to moderate IPF by slowing down the disease progression (2). Yet, these two drugs may easily cause side effects such as gastrointestinal reactions, liver dysfunction, photosensitivity and diarrhea, leading to the median survival time remains 2–5 years (3). To date, there is an urgent need to develop novel anti-fibrotic therapies capable of suppression of progression of IPF with minimal side effects.

The strategy exploiting immune systems for drug delivery is an area of great interest, as immune cells such as macrophages, neutrophils, and dendritic cells could sensitively respond to the complex inflammatory microenvironment at acute injury sites (4). These white blood cells can efficiently sense chemokines and cytokines factors and can be recruited into the inflamed tissues or/and sites (4, 5). Inflammation and immune disorders are considered as the main factors in the initiation and development of pulmonary fibrosis (6); in particular, alveolar macrophages or their prototype monocytes, especially can mediate and respond to the fibrosis related inflammatory stimulus or immune factors, such as interleukin 6 (IL-6), transforming growth factor-β (TGF-β), arginase-1 (Arg-1), etc. (7, 8). In addition, macrophages also can migrate to inflammation sites (9). These unique properties make macrophages a potential vehicle for pulmonary fibrosis drug delivery.

Depending on the local microenvironment on the fibrotic stage, macrophages can be replaced with classical activation (M1) and alternative activation (M2) phenotypes (10). In general, M1 type macrophages are responsible for wound healing after alveolar epithelial injury by secreting pro-inflammatory cytokines such as interleukin 6 (IL-6), promoting the elimination of foreign pathogens and inducing of inflammatory injury (11), while M2 type macrophages express transforming growth factor (TGF-β1), arginase-1 (Arg-1) and interleukin 10 (IL-10), which play the role of anti-inflammation, pro-angiogenesis and tissue repair (12). These anti-inflammatory cytokines also stimulate fibroblast proliferation and collagen production, which is important in the healing process, but increased expression of fibrotic factors, subsequently induced pulmonary fibrosis (13, 14). Both excessive M1 and M2 macrophages play dominant roles in IPF progression, making them promising targets via modulating macrophage polarization to reduce the level of fibrosis (15).

Exploiting macrophage for drug delivery causing anti-inflammatory response has previously been demonstrated in tissues other than the lung. Zheng et al. used tungsten oxide (WO) and indocyanine green (ICG) to construct nanoparticles. After macrophages swallowed the nanoparticles, the cells were injected through the tail vein to make the cells reach the tumor site and then given near-infrared irradiation which kill the engulfed tumor cells by photothermal effect (16). Zhang et al. designed a silica-based drug nanocapsule, which reached 16.6 pg of doxorubicin (Dox) loading per macrophage cell, allowing macrophages to reach the tumor without affecting the speed of cell migration. The drug is released after the site, which improves the utilization of the loaded drug (17).

It is worth noting that there are no reports of using macrophages as a delivery system for the treatment of pulmonary fibrosis. Moreover, no studies have reported the effect of drug-containing nanocarriers on the phenotypic changes of macrophages during the treatment of diseases. Here, we report a macrophage-mediated drug delivery system, which achieve efficient and accurate pulmonary drug delivery, with improved bioavailability of anti-pulmonary fibrosis drug dexamethasone (Dex) into the lung. Results showed that Dex decorated with liposomes (Dex-L) increased the loading efficiency of Dex into macrophages, similarly it had a bidirectional regulation effects to the phenotype when responding with the polarized macrophages which exacerbate the lung damage during the process of fibrosis. It was proved that Dex-L could keep the phenotype of the carrier itself in a balanced and non-pathogenic state, which also provided an explanation for the mechanism of Dex against pulmonary fibrosis. The therapeutic effects were further evaluated in a co-culture cell model of macrophages and fibroblasts and a mouse model of bleomycin-induced pulmonary fibrosis. We found that macrophages as a delivery system could effectively deliver Dex to the lungs with little systematic toxicity and exhibited excellent ability to inhibit pulmonary fibrosis. It is expected that this approach may provide an improved platform for targeted delivery of anti-fibrotic drugs while minimizing the problems of side effects.

## Materials and Methods

### Materials

1, 2-dioleoyl-sn-glycero-3-phosphocholine (DOPC), dioleoyl phosphoethanolamine and (DOPE), cholesterol (CHO) were purchased from Avanti Lipid (Alabaster, AL, USA). All antibodies, including Alpha smooth muscle actin (α-SMA) mouse monoclonal, rat anti-F4/80 antibody, Rabbit anti-CD206 antibody, Alexa Fluor 488-conjugated goat anti-rabbit antibody, and Alexa Fluor 647-conjugated goat anti-rat antibody, were purchased from Abcam (Cambridge, MA, USA). BLM-A5 hydrochloride was purchased from Dalian Meilun Biotech (Dalian, China). Lipopolysaccharide (LPS) was purchased from Sigma-Aldrich (St. Louis, MO USA). IL-4 was purchased from PeproTech (Rocky Hill, USA). TGF-β1 was purchased from R&D system. The ELISA test kit for IL-6, TGF-β1, IL-10, IL-1β, and Arg-1 were purchased from Zhuo Cai Biological Co., Ltd. (Shanghai, China). Nitric oxide synthase test kit was purchased from Biyuntian Co., Ltd. (Beijing, China). Masson's trichrome assay kit were purchased from Solarbio (Beijing, China). Sirius red reagent were purchased from Yuanye Bio-Technology (Shanghai, China). 3-(4,5-Dimethylthiazol-2-yl)-2,5-diphenyltetrazolium bromide (MTT) was purchased from Solarbio (Beijing, China). Dulbecco's Modified Eagle's Medium (DMEM) and other cell culture supplies were obtained from Gibco (Grand Island, NY, USA). Hoechst was provided by Invitrogen Co. (USA). Four percentage formalin was purchased from Weiao Biological (Shanghai, China).

### Fabrication of Dexamethasone-Loaded Liposomes

Dissolved DOPC (1,2-dioleoyl-sn-glycero-3-phosphocholine), cholesterol and DOPE (1,2-Dioleoyl-sn-glycero-3-phosphoethanolamine) in a molar ratio of 2:1:1 in a mixed solvent of chloroform-methanol (1:1, v/v) and dexamethasone dissolved in methanol with molar ratios of 1:1 in a 20 mL glass to form a film under nitrogen, respectively. Then the PBS (Phosphate Buffered Saline) was added to form a lipid suspension and ultrasonicated in a water bath at temperature 55°C for 30–40 min.

### The Characterize of Dexamethasone Liposomes by Dynamic Light Scattering

The diameter distribution and zeta potential of different ratios of lipids and dexamethasone were determined by dynamic light scattering (DLS) using a Malvern Zeta Sizer Nano series (Malvern ZEN3600, Malvern, UK).

### Cell Culture and Macrophage Polarization

Mouse macrophage RAW264.7 cell line and NIH-3T3 mouse fibroblast cell line were purchased from ATCC. Cells were maintained in Dulbeco's Modified Eagle's Medium (DMEM, Gibco) containing 10% (v/v) fetal bovine serum (FBS, Gibco, 10099-141), 100 Units/mL penicillin and 100 mg/ml streptomycin at 37°C in a humidified atmosphere of 95% air with 5% CO_2_. For the macrophage polarization, M1 macrophages were obtained by lipopolysaccharide (LPS, 100 ng/mL, Sigma-Aldrich Chemical Company) treatment for 12 h (18) and M2 macrophages were obtained by IL-4 (20 ng/mL, PeproTech) treatment for 48 h as previously described (19).

### Toxicity Assay

The RAW264.7 cells were seeded into 96-well plates at a density of 3 × 10^4^ cells/mL per well for 24 h before treatment and then incubated for another 24 h in the presence of different concentrations of Dex-L. The cell viability was measured using the MTT [3-(4, 5-dimethylthiazol-2-yl)-2, 5 diphenyl tetrazolium bromide]. After discarding the supernatant, each well was added 10% MTT and incubated for 4 h in the dark. The MTT solution was removed and then the formazan solution was added and shaking for 10 min. The absorbance was measured at 490 nm with a Microplate reader (Tecan, Groedig, Austria).

### Determination of Drug Loading and Release From Macrophages

Dexamethasone was labeled with FITC (Fluorescein Isothiocyanate), a fluorescein and the drug loading of macrophages could be determined by the fluorescence intensity of FITC-labeled dexamethasone. Macrophages were incubated with FITC fluorescence-labeled dexamethasone liposomes and Triton X-100 was used to disrupt the cell supernatants at 0, 2, 4, 6, and 8 h. Following the fluorescence intensity of cell lysis solutions with different times were detected at excitation wavelength of 490 nm and emission wavelength of 515 nm. After macrophages were incubated with FITC fluorescence-labeled dexamethasone liposomes for 4 h, the liquid from the wells were removed. The phenol-free red blood cell culture medium containing 1% serum was added and the fluorescence intensity of supernatants at 0, 2, 4, 6, 8, and 10 h were tested under the same fluorescence detection conditions.

### Co-culture Experiment

In our experiment, we used 24 mm Transwell® with 8 μm Pore Polyester Membrane Insert from Corning Company. The 3T3 cells were implanted in a 24-well plate. Before co-culture, macrophages and dexamethasone liposomes were allowed to interact 4 h in advance to complete the dexamethasone liposome payload. Then, the chamber was put into a 24-well co-culture plate and TGF-β1 (5 ng/mL) was added to the medium to induce the migration and activation of fibroblasts (20). Another co-culture method is collecting and resuspending the drug-loaded macrophages, they were added to a 24-well plate and co-cultured with 3T3 cells, and TGF-β1 was added to the medium to induce fibroblast activation (21).

### Scratch Assay

Draw three straight lines after the 24-well plate, and insert the cells into the well plate at a suitable density. After the cells adhere to the wall, use a 200 μL pipette to make a scratch perpendicular to the three straight lines. Images were taken with an inverted microscope (LEICA DMi8, Germany) to record cell migration at 0, 24, and 48 h.

### Immunofluorescence

For immunofluorescence staining, cells were fixed with 4% paraformaldehyde and permeabilized with 0.5% Triton X-100 for 10 min. Subsequently, the cells were treated with 2% BSA at 37°C for 1.5 h and incubated with the indicated antibodies at 4°C overnight. After being washed 3 times in PBS, the cells were stained with the corresponding secondary antibodies Alexa Fluor 488-conjugated rabbit anti-mouse antibody. Nuclei were stained with Hoechst 33342. Images were obtained with Operetta High Content Analysis (HCA) System (PerkinElmer, Boston, MA, USA). Quantitative analysis of fluorescence by ImageJ.

### Fabrication of Nanoparticle-Laden Macrophages

Macrophages were incubated with dexamethasone liposomes for 4 h and the concentration of dexamethasone was 100 μM. The macrophages were gently scraped off with a cell scraper and resuspended in PBS. Then the cells were injected into mice via tail vein.

### Animal Experiments

Idiopathic pulmonary fibrosis was established in mice model by intratracheal instillation of bleomycin (BLM)-A5 hydrochloride (Dalian Meilun Biotech, Dalian, China) (1.5 mg/kg dissolved in PBS). Mice were frequently examined by micro-CT after the treatment of BLM for 5th days. Mice presenting fibrosis lesions involving more than 25% of the lung were further divided into five experiment groups (6 mice per group), treating once daily with 3 mg/kg Dex (National Institutes for Food and Drug Control, Beijing, China). All the mice were administered via intravenous injection. Micro-CT scans were performed at baseline, then to evaluate the effect of these treatments. Lung tissues were quartered and processed for the following experiments: the left lobe was inflated and fixed in 10% buffered formalin for histological and immunohistochemical examination, and the remaining lobes were stored at −80°C and used for preparation of whole lung tissue protein extracts.

### Enzyme-Linked Immunosorbent Assay (ELISA)

The cell supernatant and mouse serum were centrifuged at 3,000 rpm for 10 min to be collected. The levels of TGF-β1 and IL-6 in the mice serum and cell-free supernatants were measured using the ELISA kits (Shanghai, China) according to the manufacturer's instructions and the cytokine concentrations were calculated using standard curves.

Mice lung tissues (50 mg) were homogenized, centrifuged and the supernatant was collected. Blood was collected from eyeball venous plexus of mice, and the serum stored at 4°C. IL-6, TGF-β1, IL-1β, and Arg-1 were detected by ELISA kits (Shanghai, China) according to the manufacturer's instructions.

### Inflammatory Cell Analysis in Whole Blood

The whole blood was obtained from the eye orbit of mice and stored in an anticoagulant tube containing EDTA. Neutrophils and lymphocytes were detected using a blood analyzer (Premier 3000, USA).

### Bronchoalveolar Lavage Fluid Collection

Mice were anesthetized by Tribromoethanol; a small incision was made on the trachea and BAL fluid was collected by cannula. Briefly, cannula was inserted into trachea with sufficient ice-cold PBS (0.5 mL each time) to collect BALF. The procedure was repeated thrice, and 70–80% recovery of collected BALF was observed. After collection of BALF, Later, BAL fluid was centrifuged at 4,000 rpm for 10 min at 4°C, and supernatant was used for determination of IL-6, TGF-β1, and IL-1β levels in BALF were detected by ELISA.

### Immunohistochemistry, Hematoxylin-Eosin Staining (H&E), and Masson's Trichrome Stain

Immunohistochemistry was performed on 4 μm, paraffin embedded lung tissue and mounted on polylysine-coated slides. The slides were cleared of paraffin and subjected to antigen retrieval (10.2 mM sodium citrate, 0.05% Tween 20, pH 6.0, 10 min). Next, quenching of endogenous peroxidase activity was achieved by incubation with 3% (v/v) H_2_O_2_ for 10 min, followed by incubation with rabbit anti-CD206 at 4°C overnight. In addition, the slides were stained with H&E for structured observation, or with Masson's trichrome stain for detection of collagen deposits according to the instructions by the manufacturer.

### Measurement of Hydroxyproline

The measurement of hydroxyproline was conducted with a hydroxyproline measurement kit (NanJing JianCheng Bioengineering Institute) according to the manufacturer's instructions. Approximately 30 mg (wet weight) lung tissue was collected. One milliliter of alkaline hydrolysate was added and the tissue was boiled at 95°C for 20 min with constant mixing. The pH was adjusted to 6.0–6.8 using the reagent provided. About 3–4 mL of supernatant was collected for measurement after sorption onto active carbon. The hydrolysate was centrifuged at 3,500 rpm for 10 min. One milliliter of supernatant was then carefully taken for measurement according to the manufacturer's instructions.

### Statistical Analysis

All analyses were performed using SPSS 7.0 software (SPSS Inc., Chicago, IL, United States) or GraphPad (GraphPad Prism 5, San Diego, CA, United States). The results were expressed as the mean ± standard deviation. Multi-group comparisons of the means were carried out by one-way analysis of variance (ANOVA) test with *post-hoc* Tukey's-test. The statistical significance for all tests was set at *P*-values of <0.05.

## Results

### Preparation and Characterization of Dex-L-MVs

**Scheme 1:**
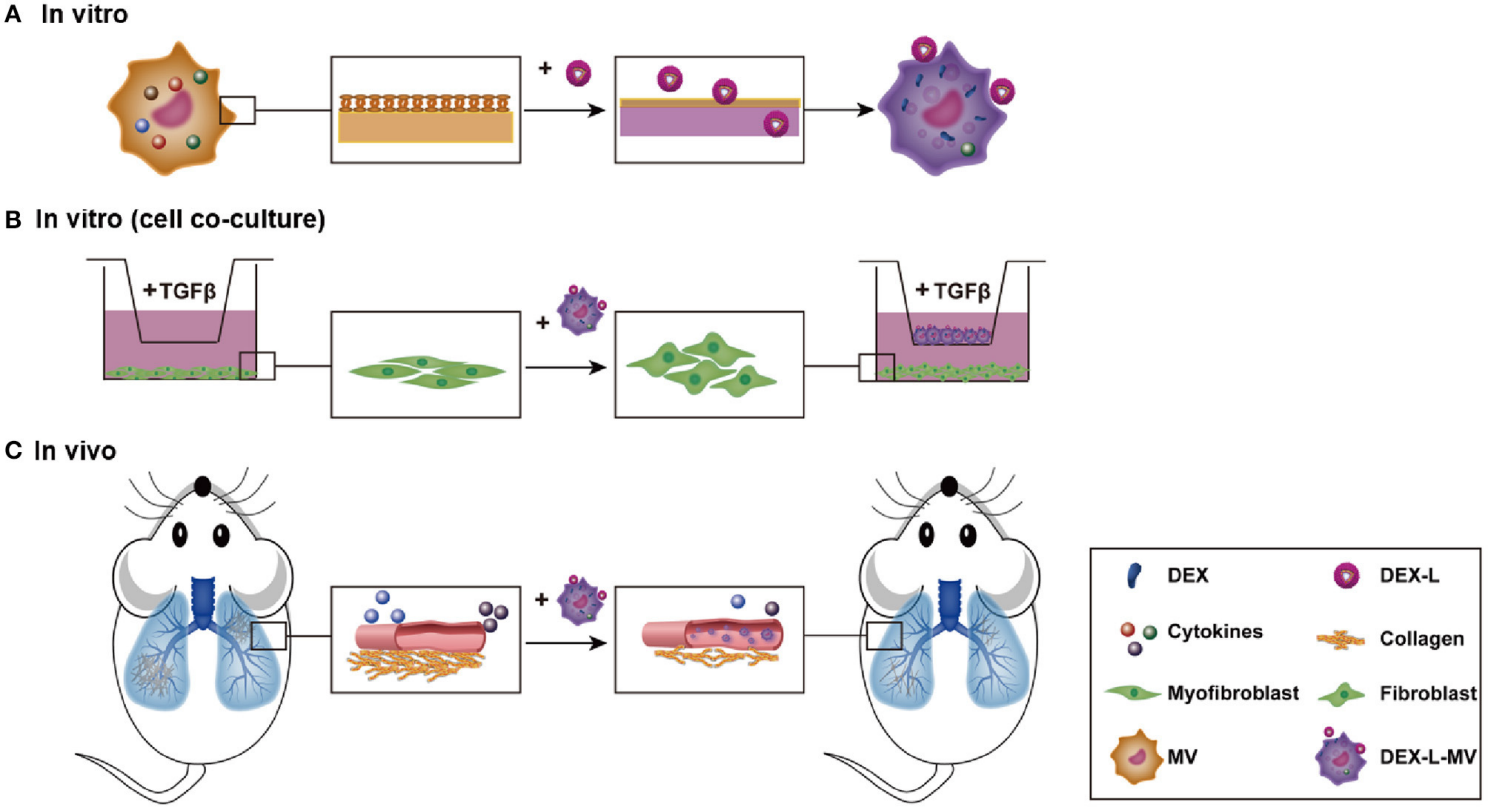
Drug delivery by macrophage targeted inflammatory microenvironment *in vitro* (**A**, cell level, **B**: co-culture level) and *in vivo* **(C)**.

Our objective is to use macrophage loaded drug-liposome to target the inflammatory micro-environment in the pulmonary fibrosis and release the drug *in situ* (Scheme 1). First, the prepared dexamethasone liposomes (Dex-L) were taken-up by macrophages in the first few hours, forming the macrophage delivery system (Dex-L-MVs). To evaluate the effect of Dex-L to the inflammatory environment, Dex-L were incubated with polarized macrophages including M1 and M2 phenotype macrophages (Scheme 1A), Dex-L-MVs further seeded with TGFβ1-induced fibroblasts to show its affect during the cell interactions and activation process (Scheme 1B). Moreover, Dex-L-MVs as a drug delivery system were injected into bleomycin-induced pulmonary fibrosis mouse model via the tail vein, and evaluated for how it regulates the development of IPF and affects the immune microenvironment (Scheme 1C).

**Figure 1:**
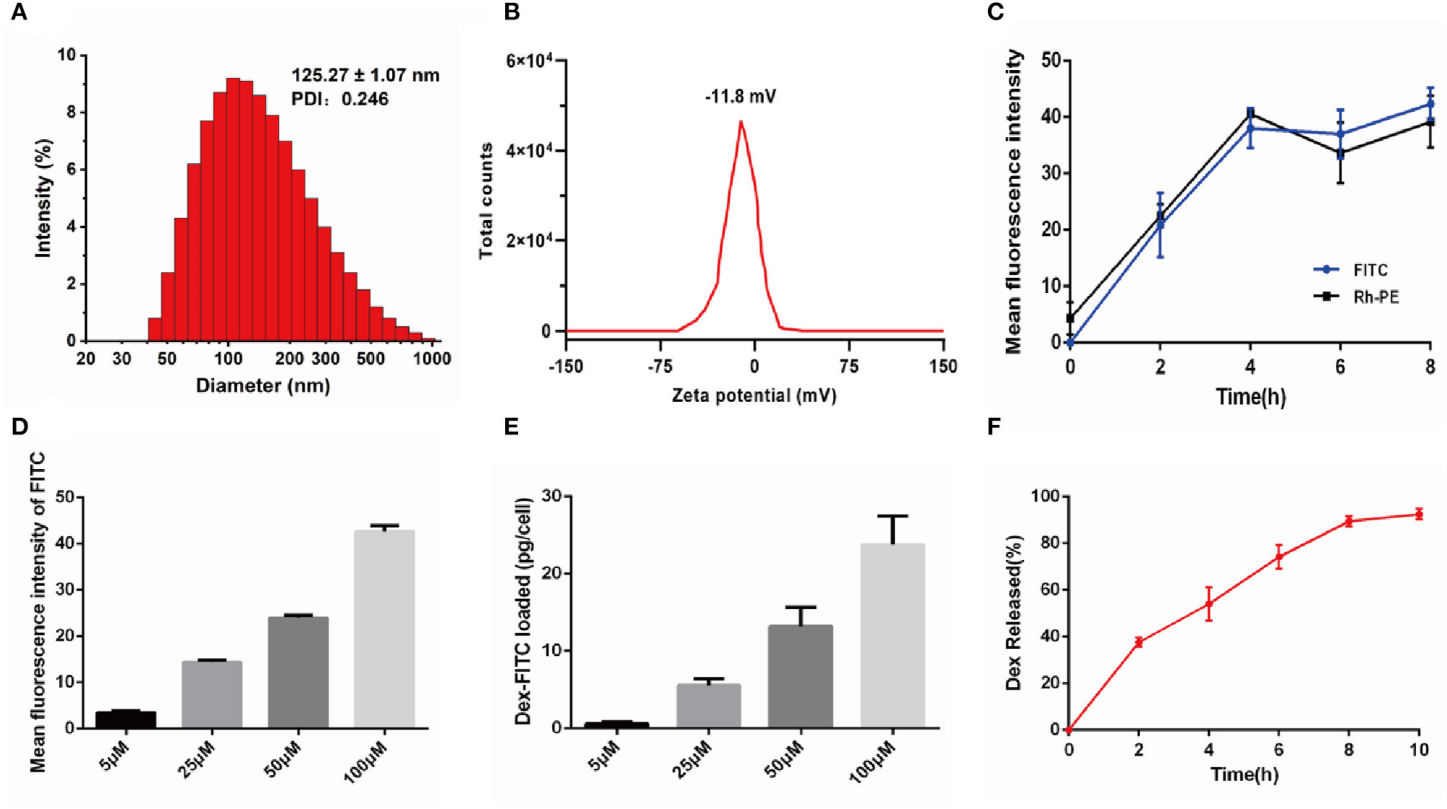
Characterization and preparation of Dex-L and Dex-L-MVs. **(A)** The size distribution and **(B)** Zeta potential of the Dex-L measured by dynamic light scattering. **(C)** The uptake of Dex-FITC at a concentration of 100 μM after different incubation times with macrophages. **(D)** Mean fluorescence intensity of FITC fluorescently labeled Dex at different concentrations after incubating for 4 h with macrophages. **(E)** Intracellular Dex-FITC contents measured when the initial FITC fluorescently labeled Dex at the concentrations of 5, 25, 50, and 100 μM. **(F)** Percentage of Dex-FITC released from Dex-L-MVs at different time points. Mean values of three independent experiments with *n* = 6 cultures per group and experiment.

Liposomes encapsulated dexamethasone (Dex-L) was swallowed and prepared by the thin film dispersion method, the particle size of Dex-L measured by dynamic light scattering is 125.27 ± 1.07 nm (Figure 1A), and The ζ-potential of the Dex-L under physiological conditions was determined as −11.96 ± 0.28 mV (Figure 1B). To address the potential toxicity of Dex-L toward macrophages, cell viability was determined in RAW264.7 cells. MTT results showed that RAW264.7 cells well-tolerated Dex-L up to 100 μM after 24 h incubation (Supplementary Figure 1).

Next, in order to measure the drug loading efficacy, RAW264.7 cells were treated by the FITC fluorescence-labeled dexamethasone (f-Dex) and Rh-PE fluorescence-labeled liposomes for different times. After measuring the fluorescence intensity of FITC and Rh-PE by Operetta High Content Analysis (HCA) System, we found that macrophages were incubated with f-Dex-L for 4 h leading to the highest effective intracellular uptake of Dex, while prolonged incubation time only slightly increased uptake of Dex (Figure 1C and Supplementary Figure 2).

The macrophages were incubated with liposomes containing f-Dex at different concentrations and the drug uptake of macrophages was concentration dependent (Figure 1D and Supplementary Figure 3). The uptake of macrophages at 4 h was about 2, 6, 13, and 25 pg/cell with the f-Dex-L at the dosing concentrations of 5, 25, 50, 100 μM, respectively (Figure 1E). Subsequently, f-Dex at the concentration of 100 μM released into macrophages was detected. Result showed that Dex released from macrophages was increased in a time-dependent manner reaching nearly 90% within 10 h (Figure 1F).

### Dex-L Bidirectionally Regulates the Macrophages Polarization

Polarized macrophages (M1 and M2 macrophages phenotypes) play important roles during the development of fibrosis. To investigate the effects of Dex-L on the polarization status of macrophages, Dex-L were incubated with M1 and M2 macrophage and subsequently cytokine cues such as nitric oxide synthase (iNOS), IL-6 and IL-10 were determined.

**Figure 2:**
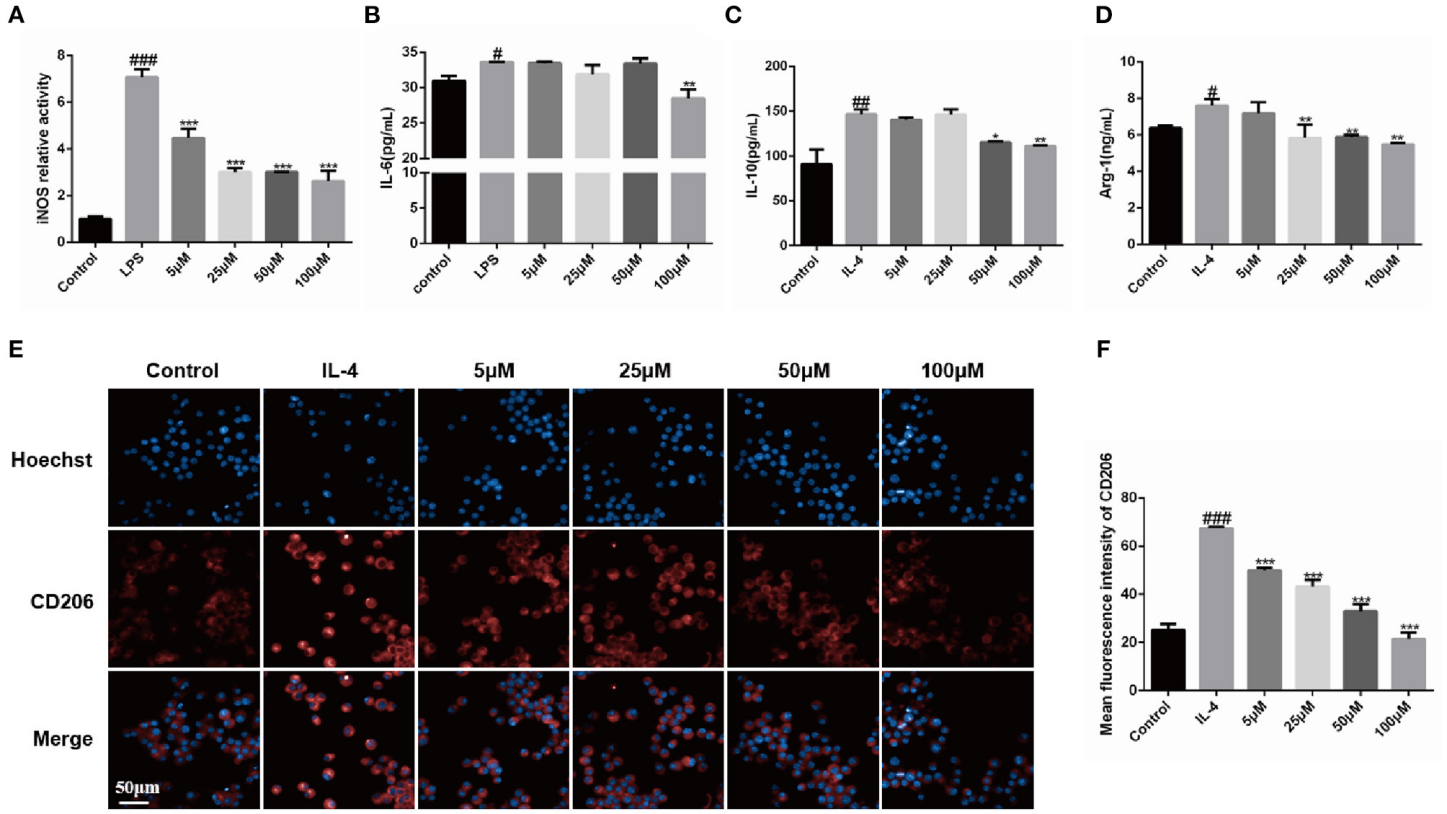
Bidirectional regulation functions of Dex-L on polarized macrophage phenotypes. The iNOS **(A)** and IL-6 activity **(B)** of LPS-induced macrophage was down-regulated by Dex-L. The secretion of IL-10 **(C)** and Arg-1 **(D)** induced by IL-4 were decreased by Dex-L. **(E,F)** The expression and the qualification of fluorescence of CD206 in IL-4 induced macrophage. Scale bar = 50 μm. Values shown are mean ± S.D. ^#^*p* < 0.05, ^##^*p* < 0.01, ^###^*p* < 0.001, vs. control group, *p < 0.05; **P < 0.01, ***P < 0.001, vs. Model group. Mean values of three independent experiments with *n* = 6 cultures per group and experiment.

As shown in Figures 2A,B, iNOS and IL-6 activities of macrophages in the group treated with 100 μM Dex-L was reduced by 63% (2.63 ± 0.45, *p* < 0.001) and 15% (28.5 ± 1.30, *p* < 0.05) respectively, indicating the M1 polarization was inhibited. Figures 2C,D shows the secretion of IL-10 and Arg-1 was also inhibited by Dex-L in a concentration-dependent manner. The expression of IL-10 and Arg-1 in the Dex-L administration group of 50 μM were down-regulated by 21.3% (115.30 ± 0.91, *p* < 0.01) and 22.6% (5.89 ± 0.10, *P* < 0.05) respectively. Figures 2E,F furtherly showed that quantitative fluorescence results of CD206 in the Dex-L administration group of 100 μM was 3-folds less than the M2 polarized group (67.40 ± 0.58 vs. 21.52 ± 2.64, *p* < 0.001). The liposomes without drug at a concentration of 100 μM sharply reduced the expression of CD206 in macrophages, indicating the inhibition effect during the process of M2 macrophage polarization (Supplementary Figure 4). All these striking results demonstrated the bidirectionally regulation functions of Dex-L on polarized macrophages, which reflecting in the down-regulation of pro-inflammatory and anti-inflammatory factors, simultaneously preserving a balance in the secretion of cytokines. The special regulation could exert a positive effect in the progress of fibrosis.

### Dex-L-MVs Inhibit the Migration and Activation of Fibroblasts in a Co-culture System

**Figure 3:**
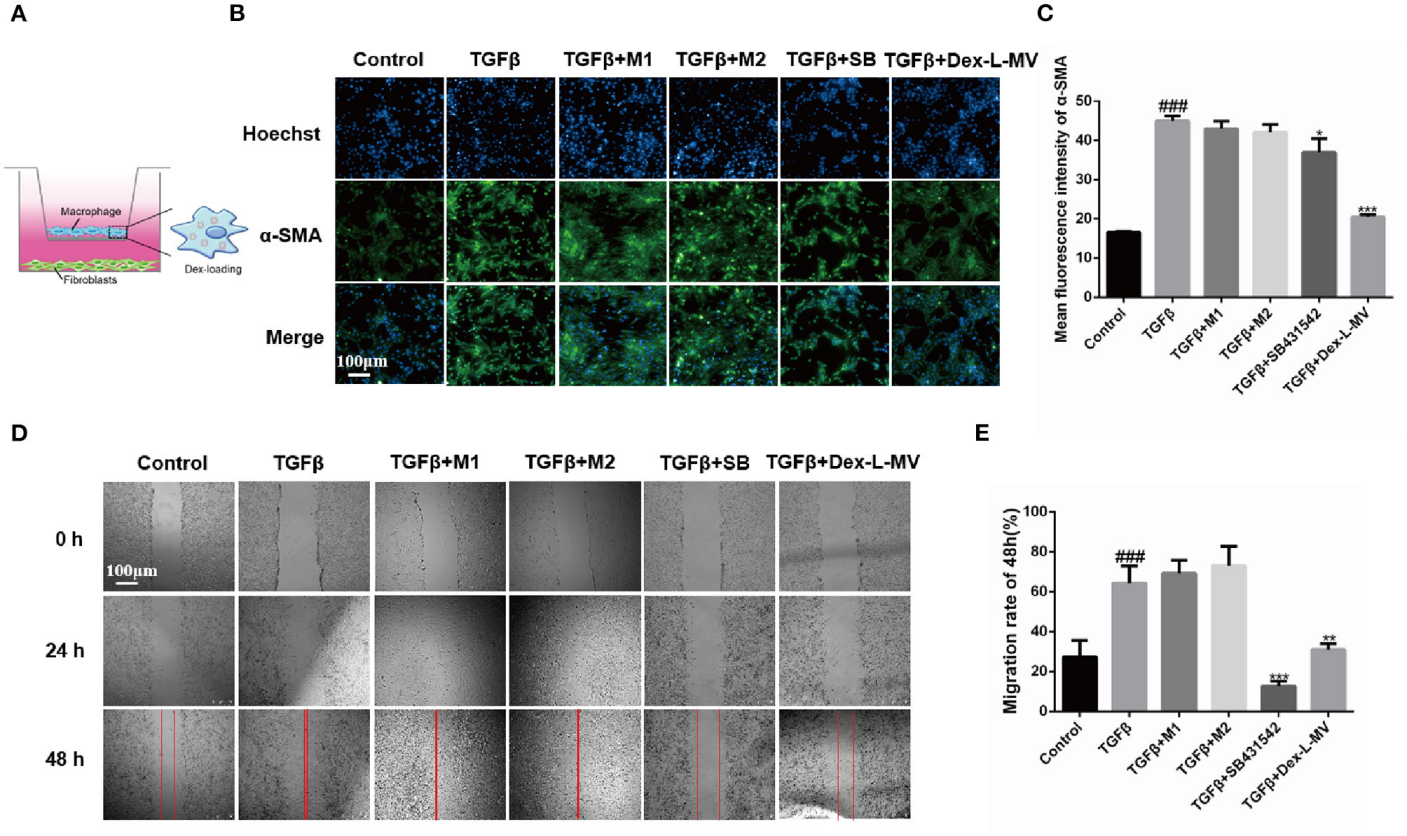
Effect of Dex-L-MVs on TGF-β1-induced NIH-3T3 in co-culture system. **(A)** A schematic of co-culture system in Transwell. **(B)** and **(C)** Expression and the qualification of α-SMA of fibroblasts with Dex-L-MVs. Scale bar = 100 μm. **(D,E)** Inhibition of migration and the qualification of fibroblasts by Dex-L-MVs. Values shown are mean ± S.D. ^###^ *p* < 0.001, vs. control group, *p < 0.05; **p < 0.01, ***p < 0.001, vs. Model group. All experiments were performed in triplicate, each being repeated at least three times.

In order to evaluate the anti-fibrotic effect of Dex-L-MVs, we constructed a co-culture system of NIH-3T3 murine fibroblast cells and RAW264.7 macrophages (21). During the process of pulmonary fibrosis, the accumulation and activation of fibroblasts into myofibroblasts is largely responsible for the collagen production within alveolar structures (22). Here, NIH-3T3 fibroblasts were seeded in the lower chamber of a 24-well plate and the macrophages pre-loaded with Dex-L were co-cultured in the migration chamber (Figure 3A). As shown in Figures 3B,C, α-SMA, the activation marker of fibroblasts into myofibroblasts, was evaluated by immunofluorescence assay. Compared to activated fibroblast cells treated with TGF-β1, Dex-L-MVs showed even better inhibition of fibroblast activation (20.50 ± 0.55, *p* < 0.001) than the group treated with TGF-β1 inhibitor SB431542 (36.96 ± 3.56, *p* < 0.05). Similarly, the migration assay showed that Dex-L-MVs were capable of significant inhibition rate comparing to activated fibroblast cells (Figures 3D,E), where M1 and M2 macrophages stimulated the migration and activation of fibroblast cells (Supplementary Figures 5, 6). A 24-h migration result also concluded that M1 and M2 macrophages promoted fibroblast migration within 24 h, and it is stronger than the group induced by TGF-β1 alone (Supplementary Figure 7).

Altogether, co-culture results indicated that Dex-L-MVs had a good ability to inhibit the migration and activation of fibroblasts, further hindering the development of IPF.

### Targeted Delivery of Dex-L-MVs Into Inflammatory Lung *in vivo*

**Figure 4:**
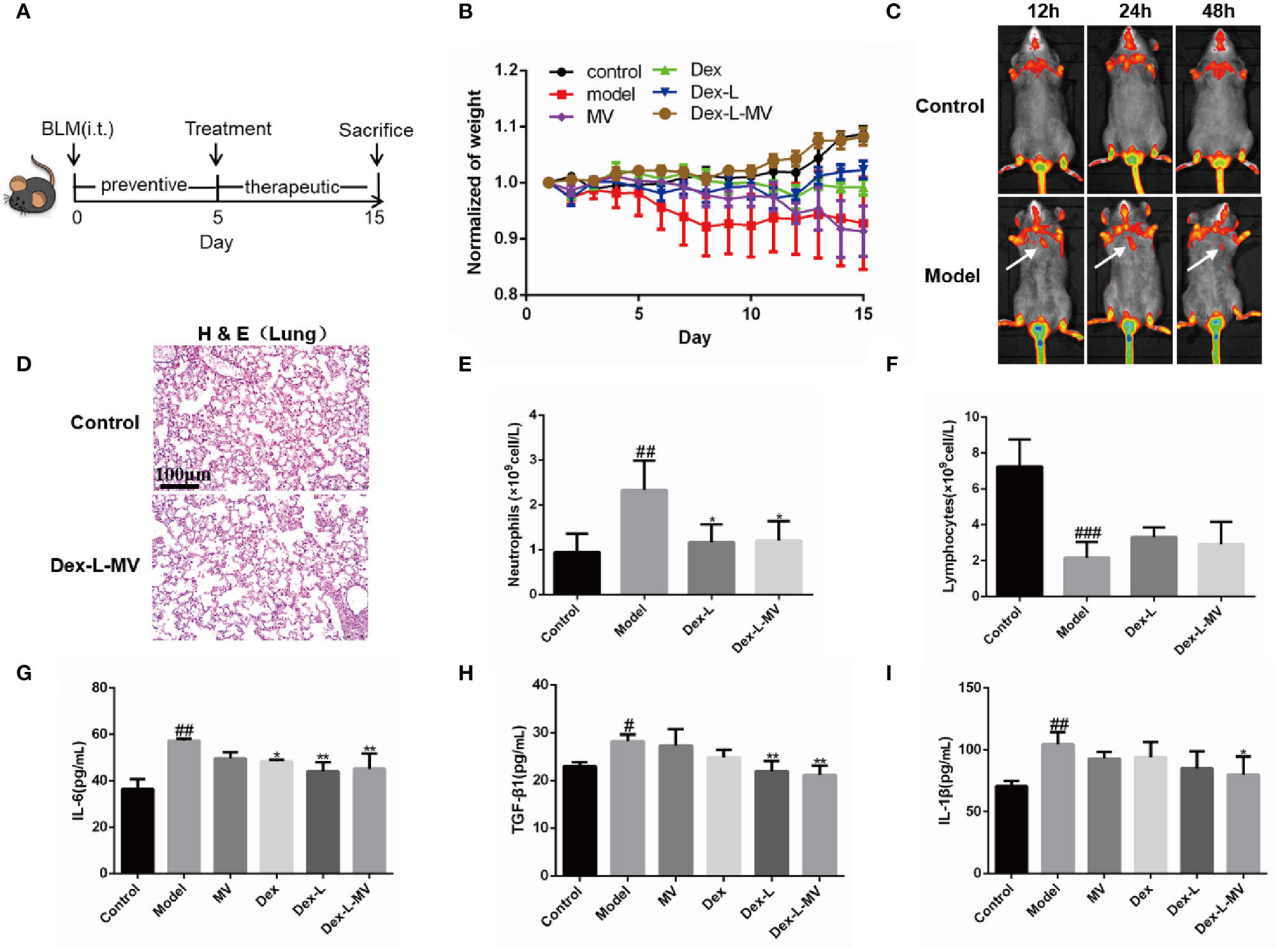
Targeted delivery of Dex-L-MVs into inflammatory lung *in vivo*. **(A)** The timeline of administration with Dex-L-MVs. **(B)** The weight change of mice (*n* = 6). **(C)** The fluorescence intensity of lipid-labeled macrophages in lung of mice over the time after intravenous injection with Dex-L-MVs. **(D)** the lung systemic toxicity of Dex-L-MVs. Scale bar = 100 μm. **(E)** The number of neutrophils and **(F)** the number of lymphocytes neutrophils per liter of peripheral blood **(G–I)** The secretion levels of inflammatory factors including IL-6 **(G)**, TGF-β **(H)** and IL-1β **(I)** in mouse BALF on the 10th day. Data representative for three independent experiments, mean ± S.D. of at least four technical replicates per time point. ^#^*p* < 0.05, ^##^*p* < 0.01, ^###^*p* < 0.001, vs. control group, *p < 0.05; **p < 0.01, vs. Model group.

A mouse model of pulmonary fibrosis was established by tracheal instillation of BLM (1.5 mg/kg) (23). We detected microCT scan imaging to allow the evaluation of lung fibrosis at different time points (0, 5, 10, 15 days) after bleomycin induction, and found that there were diffuse shadows in the lungs from the 5th day (Supplementary Figure 8). Therefore, after BLM induction on the 5th day, mice with more than 25% pulmonary fibrosis lesions were further divided into five experimental groups for the next drug treatment. The mice were sacrificed by excessive anesthesia at 15 days after BLM challenge (Figure 4A). The mean body weights of the mice in control group were slightly increased, while the weight of mice instilled with BLM significantly decreased on 5th day. Figure 4B showed that the mice administrated with Dex, Dex-L, and MVs showed weight restore, while the group with Dex-L-MVs markedly reversed these reductions. To test the delivery positions of Dex-L-MVs, we labeled liposomes with Liss Rhod-PE (Rh-PE) red fluorescence and injected macrophages that phagocytosed fluorescent liposomes into mice. We observed the fluorescence accumulation in control mice and fibrotic mice after 12 h post-treatments. As expected, stronger fluorescence accumulation (white arrows) into the pulmonary of fibrotic mice when compared with that in mice in control group, indicating that Dex-L can more efficiently reach the lungs with the help of macrophages (Figure 4C). This result proved that the inflammatory pulmonary can recruit macrophages to achieve the targeted therapeutic effect. We investigated the systemic toxicity of Dex-L-MVs toward C57 mice (*n* = 6) using hematoxylin and eosin (H&E) staining. The results showed that no pathological changes in organs after 10 days treatment, demonstrating that systemic toxicity was rarely exhibited by intravenous injection of the Dex-L-MVs groups (Figure 4D and Supplementary Figure 9). Next, we further investigated the effect of dexamethasone-liposomes infused into the lungs of mice on the regulation of immune responses. As expected, the blood routine results showed that the model group mice had up-regulation of neutrophils and down-regulation of lymphocytes, when compared to the wild-type mice. After the administration of Dex-L-MVs, the increase of neutrophils and the decrease of lymphocytes were significantly inhibited (Figures 4E,F). Moreover, after detecting the expression of inflammatory cytokines and chemokines in the collected bronchoalveolar lavage fluid, the results demonstrated that the inflammatory factors including IL-6, TGF-β1, and IL-1β were significantly decreased after the administration of Dex-L and Dex-L-MVs, indicating the abnormal fibrosis inflammatory microenvironment was well-remodeled (Figures 4G–I). Together, these results indicate the potential application of Dex-L-MVs induced targeted delivery for direct regulation of inflammatory and immune responses.

### Therapeutic Effect of Dex-L-MVs on Bleomycin-induced IPF Mice

**Figure 5:**
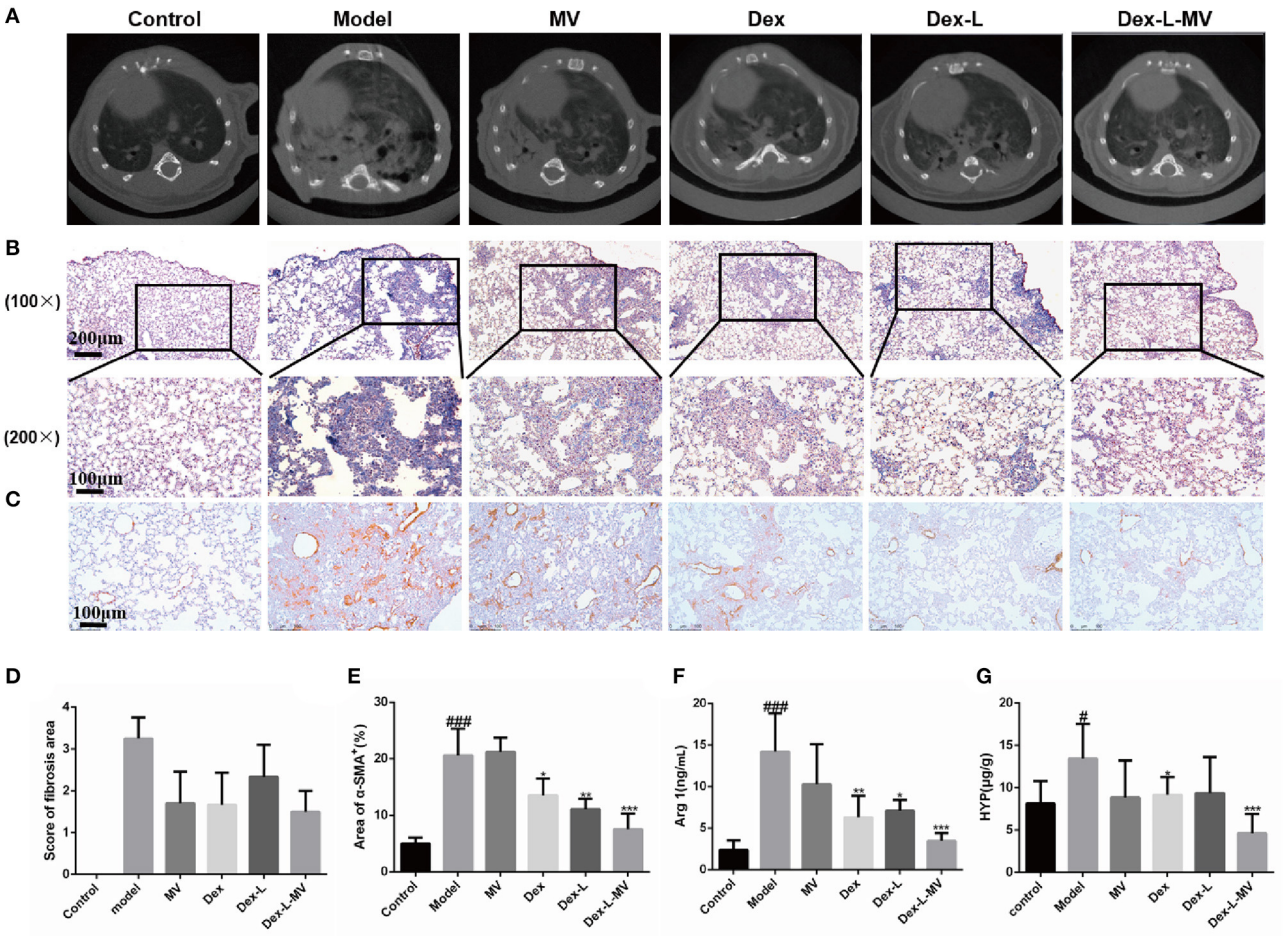
Therapeutic effects of Dex-L-MVs on bleomycin-induced mouse model of IPF. **(A)** μCT scanning images to show the pulmonary fibrotic level of different groups. **(B)** The evaluation qualification of collagen deposition by Masson's trichrome staining of lung tissue sections. The inset shows a 4x image of lung lobe **(C)** The evaluation and **(E)** qualification of α-SMA by Immunohistochemistry of lung tissue sections. **(D)** The score of μCT images according to 85% percentile density. **(F)** The content of Arg-1 expressed in lung tissues. **(G)** The content of HYP expressed in lung tissues. All experiments were performed in triplicate, each being repeated at least three times. Values shown are mean ± S.D. ^#^*p* < 0.05, ^###^*p* < 0.001, vs. control group, *p < 0.05; **p < 0.01, ***p < 0.001, vs. Model group.

A usual interstitial pneumonia pattern on high-resolution computed tomography (HRCT) is essentially diagnostic of IPF in the appropriate clinical setting. Recently micro-CT (μCT) has been used to quantify the pulmonary fibrosis in mice, and the image quality of the μCT of each mouse was assessed semi-quantitatively on a four-point ranking scale as previously described (24, 25). The μCT results in Figures 5A,D showed that mice treated with Dex-L-MVs reduced the degree of BLM-induced fibrosis, with a score of 1.5–2. Nevertheless, model group and MVs group showed increasing obscured pulmonary vessels and an abnormal bronchial wall contour, which mean ranking scale was defined around 4, the score of mice administrated with Dex or Dex-L was 2.5–3, which showed that the level of lung fibrosis was slightly reduced. Furthermore, Masso's trichrome staining in Figure 5B and Supplementary Figure 10 demonstrated that collagen deposition in model group was more than 3-folds than that in control group and the Dex-L-MVs treatment group, indicating the significant inhibition effects of Dex-L-MVs on collagen deposition in the mouse model of IPF. To investigate the effect of the drug delivery system on the activation of pulmonary fibrosis effector cell myofibroblasts, we performed immunohistochemical staining on mouse lung tissue sections and detected the expression of α-SMA, a fibroblast activation marker. The results showed that α-SMA expressed in the Dex-L-MVs group was about 3 folds lower than that in the model group (7.53 ± 2.77 vs. 20.57 ± 4.76, *p* < 0.001) (Figures 5C,E). In addition, we also detected the expression of arginine-1 (Arg-1) in lung tissue homogenate (Figure 5F). The expression level of Arg-1 in the mice from the model group was increased around 4-folds when compared with that in control group (14.22 ± 4.59 vs. 2.37 ± 1.17, *p* < 0.001), after treatment with Dex-L-MVs, the expression of Arg-1 was significantly decreased (3.49 ± 0.93 vs. 14.22 ± 4.59, *p* < 0.001), which further presented the immune-modulatory effect of our macrophage delivery system containing DEX. We also evaluated the content of hydroxyproline (HYP) in the lung tissues, and found that Dex-L-MVs sharply reduced the amount of collagen in the lungs of BLM treated mice (Figure 5G).

### Dex-L-MVs Reduces Lung Inflammation in Fibrotic Mice

**Figure 6:**
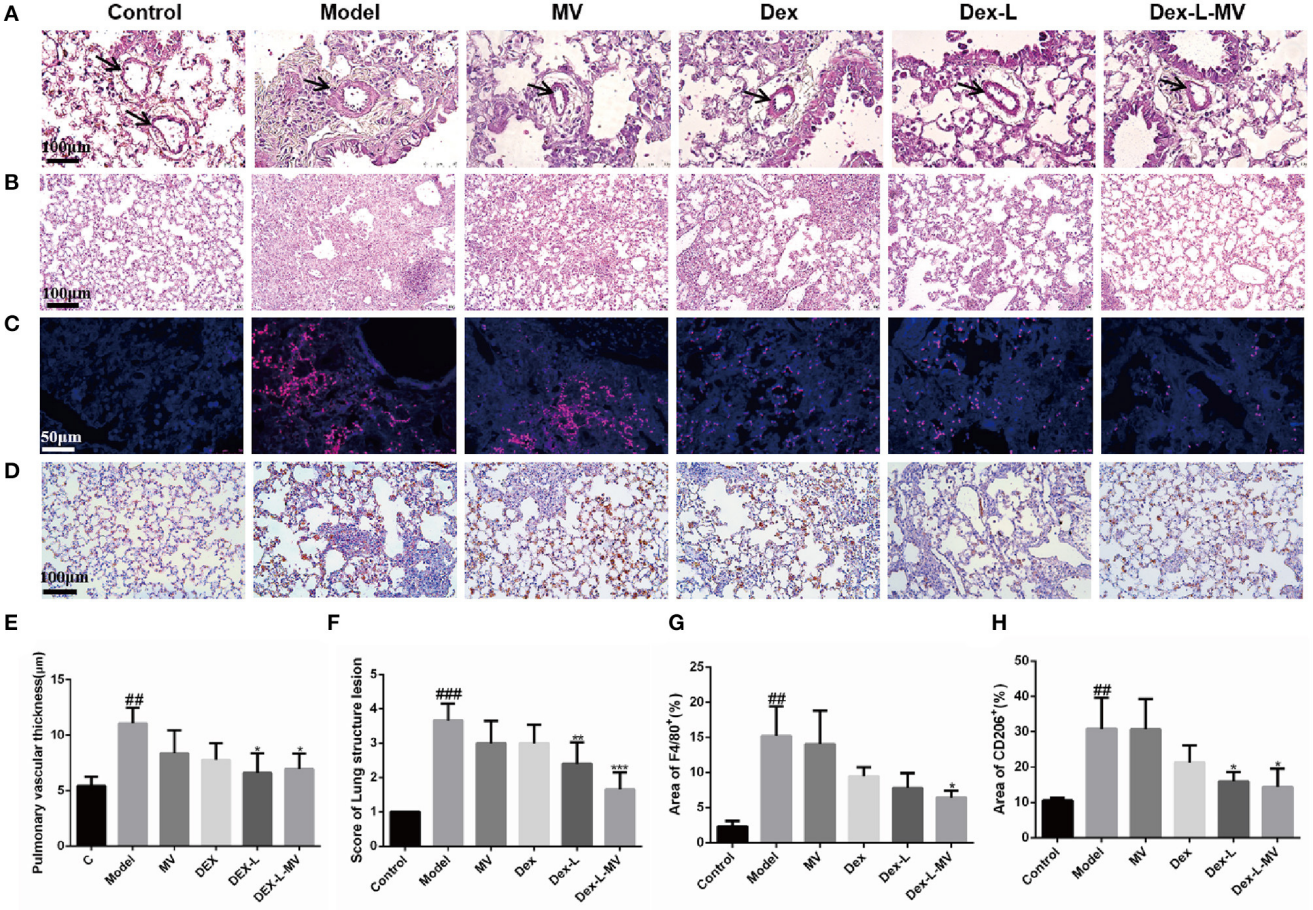
The reduction of lung inflammation in fibrotic mice with Dex-L-MVs. **(A,E)** The thickness changes of lung vascular wall with H&E staining after administration and qualification by ImageJ. Scale bar: 100 μm. **(B,F)** The inflammation and the alveolar structure of lung with H&E staining and qualification analyses. Scale bar: 100 μm. **(C,G)** The macrophage labeled with F4/80 (red channel) in lung tissue using immunofluorescence. Scale bar: 50 μm. **(D)** and **(H)** The M2 macrophage labeled by CD206 with immunohistochemistry. Scale bar: 100 μm. All experiments were performed in triplicate, each being repeated at least three times. Values shown are Representative images mean ± S.D. ^##^*p* < 0.01, ^###^*p* < 0.001, vs. control group, *p < 0.05; **p < 0.01, ***p < 0.001, vs. Model group.

During the development of fibrosis, the inflammatory response will drive the process of fibrosis (4). H&E stain of lung tissue revealed that severe pulmonary fibrosis was accompanied by a significant thickening of the blood vessel wall in the lung, which was suppressed by Dex-L and Dex-L-MVs treatment (Figures 6A,E). Mice in the model group showed severe inflammatory infiltration and destroyed alveolar structure. After Dex-L-MVs treatment, the lung inflammation in the mice was significantly reduced and the alveolar structure was significantly improved (Figures 6B,F). The occurrence of fibrosis is accompanied by large number of macrophages infiltration, especially the M2 type macrophage. In order to verify the regulatory effect of Dex-L-MVs on lung macrophage phenotype, we performed immunofluorescence staining and immunohistochemical staining on mouse lung tissue sections. Here, F4/80 was used to mark the total macrophages; the type 2 macrophages were labeled with CD206. Interestingly, Dex-L-MVs treatment not only down-regulated the total macrophages infiltration in the lung tissues of the mouse model of IPF (Figures 6C,G), but also reduced the total quantity of M2 macrophages which were marked by CD206 antibody (Figures 6D,H). These results indicate that Dex-L-MVs can improve lung fibrosis by reducing the activation of type 2 macrophages. ELISA results further indicated the expression levels of IL-6, TGF-β1, and IL-1β in serum was up-regulated in the model group compared with the normal mice, while Dex-L and Dex-L-MVs efficiently reduced their expressions (Supplementary Figure 11). Thus, macrophage delivery of Dex-L can potentially reduce the inflammatory cues and balance the immune environments *in vivo*.

## Discussion

Idiopathic pulmonary fibrosis is a chronic and progressive lung disorder, for which only two drugs, nintedanib (NDN) and pirfenidone (PFD) were clinically approved. Because of their strong side effect, there is an urgent need to develop safer therapeutic approaches to suppress the progression of IPF. So far, only few designed targeted drug delivery systems using particles for IPF are reported, all attempts in targeted drug delivery have relayed on the specific recognition to the surface receptor. For example, Chang employed matrix metalloproteinase-2 (MMP-2) responsive peptide (peptide E5)-modified engineered liposomes loaded with nintedanib (NIN) and colchicine (COL) that can firstly target endogenous monocyte-derived multipotent cells (MOMCs) and then be selectively delivered into IPF lungs (26). Liposomal quercetin could attenuate the bleomycin-induced pulmonary fibrosis *in vivo* by the suppression of inflammatory cytokines (27). These delivery system have their own limitations, including safety issues of inhaled nanoparticles or lower drug loading efficacy (28, 29).

Macrophages or monocytes can respond to chemotactic cues and migrate to inflammatory sites, making them a potentially attractive drug delivery vehicle. The macrophages have used their powerful phagocytic function to deliver drugs to the hypoxic region of tumors in the form of “Trojan horses” (30–32). During the development of pulmonary fibrosis, inflammation is also closely related (12, 33, 34), but as far as we know, there are no reports of fibrotic diseases that use macrophages as a potential drug delivery vehicle. M1 and M2 macrophages are distinct cell subtypes and are both involved in the pathogenesis of pulmonary fibrosis. Elective treatment in clinic for IPF remains a challenge due to low drug accumulation in lungs and imbalanced polarization of pro/anti-inflammatory macrophages (M1/M2 macrophages) (13, 26). Therefore, strategies aimed at modulation of lung macrophage phenotypes may have great potential for prevention and treatment of pulmonary fibrosis in clinical settings (5, 6, 9, 26).

## Conclusion

Here, we developed a macrophage-based delivery system loading dexamethasone liposomes to attenuate pulmonary fibrosis *in vitro* and *in vivo*. With the help of macrophage membranes or membrane-like membranes to specifically intervene in the immune response via the specific binding on the cell membrane (35, 36), we can targeted delivery drugs into the pulmonary sites in the BLM induced fibrosis mice. In addition, to answer the question about the regulatory effect of the drug contained in macrophages on the polarization of macrophages, we also demonstrated the interactions between macrophage polarizations and drug-liposomes, further evaluated how the delivered drugs affect immune microenvironment, in particular, the macrophages infiltrations. Dex-L can inhibit the activation of CD206-positive macrophages in lung tissue and *in vitro* experiments. Similar to M2 macrophage polarization, interestingly, Dex-L was observed to exhibit an inhibitory activity on the polarization of M1 macrophages by decreased iNOS and IL-6 levels *in vitro*. We anticipate that this cell-based drug delivery strategy will speak new *in vitro, in vivo* in the field of endogenous immune targeting IPF therapy.

## Data Availability Statement

The original contributions generated in the study are included in the article/Supplementary Material, further inquiries can be directed to the corresponding author.

## Ethics Statement

The animal study was reviewed and approved by Institutional Animal Care and Use Committee at the Tianjin International Joint Academy of Biotechnology and Medicine.

## Author Contributions

XS performed experiments, analyzed data, and wrote the manuscript. YW analyzed the data. JY designed of the study, reviewed data, and contributed to the project conception and manuscript revision. YZ, GF, and FT provided guidance and partial funding support. ZX revised the manuscript. All authors reviewed, revised, and approved the manuscript for submission.

## Conflict of Interest

The authors declare that the research was conducted in the absence of any commercial or financial relationships that could be construed as a potential conflict of interest.
