# Supplementary material for: Macrophage-Targeted Lung Delivery of Dexamethasone Improves Pulmonary Fibrosis Therapy via Regulating the Immune Microenvironment
Source: Front Immunol. 2021 Feb 18;12:613907. doi: 10.3389/fimmu.2021.613907 (PMC7935565; doi:10.3389/fimmu.2021.613907)
Supplement: Supplementary file 1 [file Data_Sheet_1.docx]

Supplementary Material

Macrophage-targeted Lung Delivery of Dexamethasone Improves Pulmonary Fibrosis Therapy via Regulating the Immune Microenvironment

Xiaoqing Sang^1,2^, Yuanyuan Wang^1,2^, Zhifeng Xue^1,2^, Dawei Qi^3^, Guanwei Fan^4,5^, Fei Tian^2,6^, Yan Zhu^1,2^, Jian Yang^1,2^*

^1^ Tianjin Key Laboratory of Chinese Medicine Pharmacology. Tianjin University of Traditional Chinese Medicine, Tianjin 300193, China.

^2^ State Key Laboratory of Component-based Chinese Medicine, Tianjin University of Traditional Chinese Medicine, Tianjin 301617, China.

^3^ Medcity Research Laboratory, University of Turku, Tykistokatu 6, FI-20520 Turku, Finland

^4^ Medical Experiment Center, First Teaching Hospital of Tianjin University of Traditional Chinese Medicine, Tianjin, China.

^5^ Tianjin Key Laboratory of Translational Research of TCM Prescription and Syndrome, Tianjin, China.

^6^ Tianjin Key Laboratory of TCM Chemistry and Analysis. Tianjin University of Traditional Chinese Medicine, Tianjin 300193, China.

*** Correspondence:**

Jian Yang

email: [yang.j2017@tjutcm.edu.cn](mailto:yang.j2017@tjutcm.edu.cn)

# Supplementary Figures

**Supplementary Figure 1.** Incubate with Dex, Dex-L and liposome for 24 hours, the effect of macrophage cell viability.

**Supplementary Figure 2.** Within 0 to 8 hours, immunofluorescence observes the uptake of Dex-L by macrophages. Dexamethasone is labeled with FITC and liposomes are labeled with Rh-PE.

**Supplementary Figure 3.** Fluorescence images of Dex-L taken up by macrophages at concentrations of 5 μM, 25 μM, 50 μM, and 100 μM.

**Supplementary Figure 4.** Immunofluorescence detected the inhibitory effect of 100μM liposomes on macrophage type 2 activation. Data are presented as mean ± SD from three independent experiments. The t-test was applied to analyze results. ^##^*p* < 0.01 vs. control group, ^**^*p* < 0.01, vs. Model group.

**Supplementary Figure 5.** Fibroblast migration in the co-culture model at 24 hours. Data are presented as mean ± SD from three independent experiments. The t-test was applied to analyze results. ^*^*p* < 0.05 and ^**^*p* < 0.01.

**Supplementary Figure 6.** In a co-culture system, immunofluorescence was used to detect the effect of macrophages of different phenotypes on NIH-3T3 activation. Data are presented as mean ± SD from three independent experiments. The t-test was applied to analyze results. ^###^*p* < 0.001, vs. control group, ^**^*p* < 0.01, ^***^*p* < 0.001, vs. Model group.

**Supplementary Figure 7.** In the co-culture system, immunofluorescence to detect the effect of different phenotypes of macrophages on the fibroblast migration. Data are presented as mean ± SD from three independent experiments. The t-test was applied to analyze results. ^*^*p* < 0.05 and ^**^*p* < 0.01.

**Supplementary Figure 8.** microCT scan imaging at different time points after bleomycin induction.

**Supplementary Figure 9.** The systemic toxicity of Dex-L-MVs.

**Supplementary Figure 10.** Scores of collagen deposition in the IPF mice. The t-test was applied to analyze results. ^###^*p* < 0.001, vs. control group, ^***^*p* < 0.001, vs. Model group.

**Supplementary Figure 11.** Detection of IL-6, TGF-β1and IL-1β factor expression in mouse serum with ELISA kit. Data are presented as mean ± SD from three independent experiments. Data are presented as mean ± SD from three independent experiments. The t-test was applied to analyze results. ^*^*p* < 0.05 and ^**^*p* < 0.01.
